# The Effect of Measles on Health-Related Quality of Life: A Patient-Based Survey

**DOI:** 10.1371/journal.pone.0105153

**Published:** 2014-09-09

**Authors:** Dominic Thorrington, Mary Ramsay, Albert Jan van Hoek, W. John Edmunds, Roberto Vivancos, Antoaneta Bukasa, Ken Eames

**Affiliations:** 1 Centre for the Mathematical Modelling of Infectious Diseases, London School of Hygiene and Tropical Medicine, London, United Kingdom; 2 Public Health England (Colindale), London, United Kingdom; 3 Public Health England (Cheshire and Merseyside), Liverpool, United Kingdom; Fondazione IRCCS Policlinico San Matteo, Italy

## Abstract

**Background:**

Measles is a highly contagious and potentially fatal illness preventable through vaccination. Outbreaks in the UK and many other European countries have been increasing over recent years, with over 3,207 laboratory-confirmed cases reported by Public Health England from January 2012 to the end of June 2013. To aid rational decision making regarding measles control versus other use of healthcare resources, it is important to measure the severity of measles in units that are comparable to other diseases. The standard metric for this in the UK is the quality-adjust life year (QALY). To our knowledge, the impact of measles on health-related quality of life (HRQoL) in terms of QALYs has not been quantified.

**Methods and Findings:**

Individuals with confirmed measles were sent questionnaires requesting information on the short-term impact of the illness on their HRQoL using the EuroQol EQ-5D-3L questionnaire. HRQoL was reported for the day the questionnaire was received, the worst day of infection and at follow-up three weeks later. 507 questionnaires were sent to individuals with confirmed measles with 203 returned (40%). The majority of respondents were not vaccinated. The mean time off work or school was 9.6 days. The mean duration of perceived illness was 13.8 days. The mean number of QALYs lost was 0.019 (equivalent to 6.9 days). The overall burden of disease in terms of QALYs lost in England based on the total number of confirmed cases in the twelve month period from 1^st^ June 2012 was estimated to be 44.2 QALYs.

**Conclusion:**

The short-term impact of measles infection on HRQoL is substantial, both at the level of the individual patient and in terms of the overall disease burden. This is the first attempt to quantify QALY-loss due to measles at a population level, and provides important parameters to guide future intervention and control measures.

## Introduction

Measles is a highly infectious notifiable disease that can be severe in infants, pregnant women and immunocompromised individuals [1], [2]. Measles is preventable through the measles-mumps-rubella vaccination programme (MMR), with measles vaccination introduced in the UK in 1968 [1]. The reported coverage is 92.9% [3] although uptake fell in the late 1990s from 92% in 1996 to 80% in 2003 [4] after the suggestion of a potential link between the vaccine and autism [5] that subsequently proved to be unfounded [6]–[8].

Previous measles outbreak reports focus on the epidemiology of the disease [9]–[11], rather than the overall disease burden in terms of health-related quality of life (HRQoL). The impact of infectious diseases on HRQoL is a developing field of research, whose aim is to express the burden of disease not only in number of cases but also in disease days and the impact of these disease days. Doing so enables a comparison between diseases and helps in the fair allocation of resources. In England the evaluation of resource allocation is formalised in cost-effectiveness analyses [12], .

A standard method to measure the disease burden is the use of quality-adjusted life years (QALYs). A QALY is a generic measure incorporating both the length of time that patients experience health reduction and the magnitude of the health reduction [14]. To calculate QALYs first the condition-specific health utilities, which give an estimate of the impact on HRQoL for the condition in question, must be established. Health utilities commonly take values between 0 and 1, corresponding to utilities for death and perfect health respectively (although some systems of measurement allow utilities of less than 0 to be reported). These utilities are used in conjunction with the duration of the health reduction to calculate the QALY-loss (and thus the potential QALY-gain for any proposed intervention or new health technology).

To our knowledge, no measure of health utilities has previously been attempted for measles, despite the global significance of this infection. This study attempts to gather health utilities specific to measles during the 2012-13 regional measles epidemics in England, as well as other direct and indirect effects of a measles epidemic on a population including symptoms during infection; disruption at home due to time off work or school for individuals with confirmed measles; hospitalisations and carer time off work.

## Methods

In this study, standardised postal questionnaires were sent to individuals with confirmed or suspected measles. Questionnaires were sent to individuals with suspected measles in the North West England outbreak from 1^st^ June 2012 and the study was extended throughout England from 2^nd^ October 2012 to 5^th^ July 2013 targeting only individuals with confirmed measles.

### Case definition

Individuals with suspected measles were confirmed positive if they were measles immunoglobulin M-positive on saliva or through polymerase chain reaction testing in urine, saliva or a throat swab. A suspected measles case was defined using the following criteria from Vivancos et al. 2012 [11]:

- Clinical presentation: fever and measles-like rash and one or more of the following symptoms: cough, conjunctivitis, coryza, or Koplik's spots.
- Residence/reported from: residence or history of travel to endemic, outbreak or adjacent areas, or being a close contact of a confirmed or probable case of measles.

### Exclusion criteria

Individuals in traveller communities with laboratory-confirmed measles were not invited to participate in the study, because Public Health England engages with this community through different protocols and procedures [15]. A member of the traveller community was defined as someone self-identifying as a member of the traveller community or someone living on a traveller site, whether authorised or not authorised.

We excluded individuals with confirmed measles with a reported symptom onset date more than two weeks before case status was confirmed to minimise the time between perceived symptom onset and receiving the first questionnaire.

Unless stated, the analysis that follows is based on individuals with confirmed measles.

### EuroQol EQ-5D-3L

The EuroQol EQ-5D-3L is a generic multi-attribute health-state classification system [16], [17]. HRQoL is assessed in five dimensions: mobility, self-care, usual activities, pain/discomfort and anxiety/depression. Each dimension is assessed using three levels: no problems, some problems and severe problems, facilitating the evaluation of 243 ( =  3^5^) different health states. The EuroQol scoring algorithm converts the responses into a health utility specific to the individual's health state. A visual analogue scale (VAS) invites the individual to rate their health state on a scale from 0 – 100, with 0 being the worst health state imaginable and 100 being the best health state imaginable. The National Institute for Health and Clinical Excellence recommends the EuroQol EQ-5D-3L for use in cost-effectiveness analyses in the United Kingdom [13].

Three age-specific EQ-5D-3L questionnaires were used: the standard EQ-5D-3L for all individuals aged 13 years and older; the child-friendly EQ-5D-Y for all individuals aged between 7 – 12 years [18] and a proxy version of the standard EQ-5D-3L for individuals aged less than 7 years to be completed by the child's parent or guardian. All three versions of the questionnaire use both the same algorithm and scoring tariff to convert responses into health utilities.

One year is equivalent to 365 days; therefore 1 QALY would be equivalent to 365 quality-adjusted life days (QALDs). The QALD has previously been used to report the impact of influenza on HRQoL [19], and for ease of interpretation we express loss of HRQoL in terms of QALDs below.

### Questionnaires

Individuals were sent an initial questionnaire requesting details of their illness and its impact on their HRQoL for both the worst day of infection and the day that the questionnaire was received using the EQ-5D-3L. Three weeks later they were sent a follow-up questionnaire to obtain a further HRQoL measurement at recovery. Individuals who did not return the first questionnaire were sent it a second time along with the follow-up questionnaire three weeks later. We assumed that a three week period was sufficient for typical symptoms of measles to subside [20], and we assumed that if individuals reported that they had recovered then they were no longer suffering a measles-related reduction in their HRQoL. The value of HRQoL reported by individuals who reported having recovered was treated as their baseline HRQoL for the purposes of calculating QALY loss.

**Figure 1:**
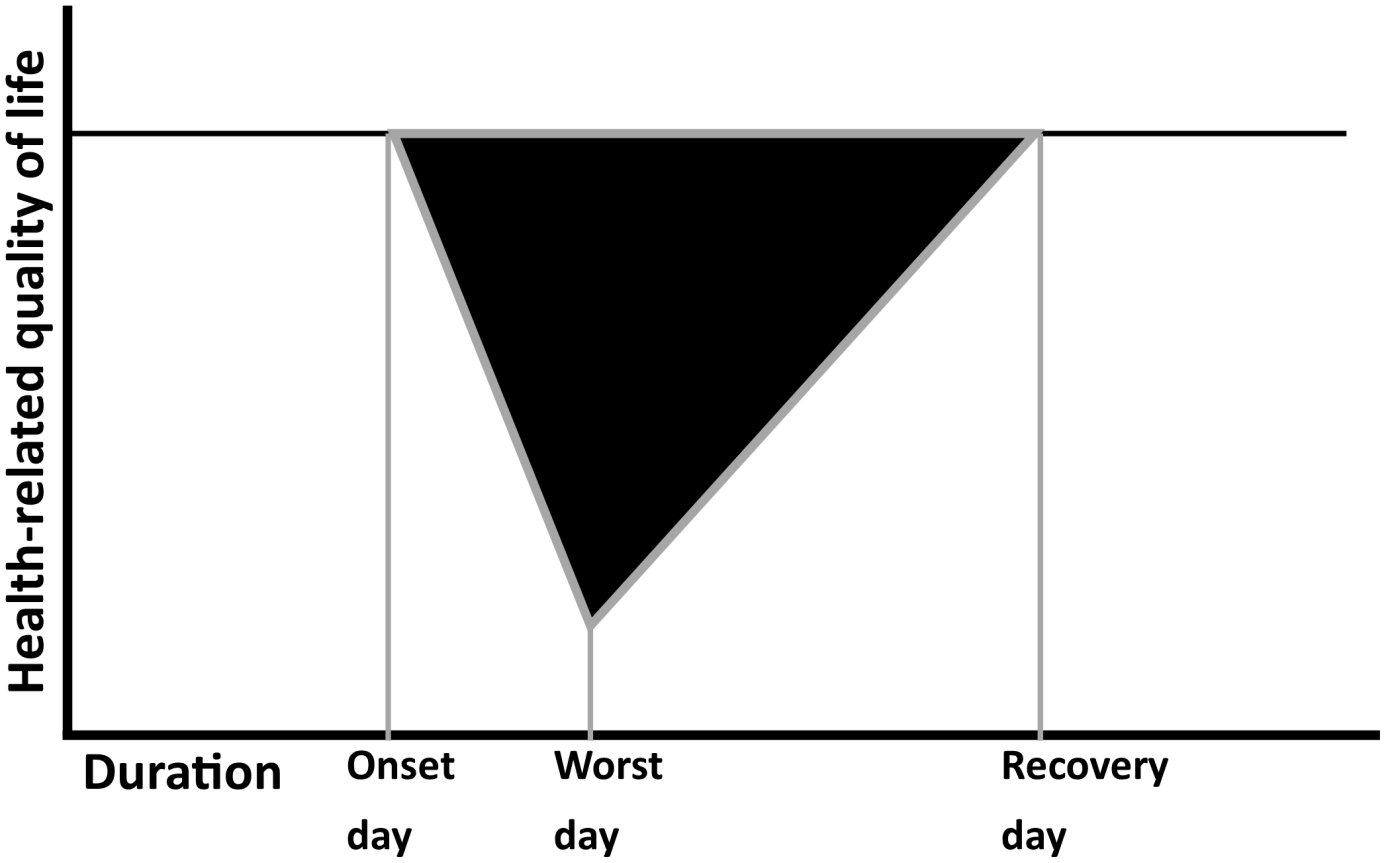
Illustration of the calculation of short-term loss of health-related quality of life (HRQoL). HRQoL is plotted against time; the area of the black triangle represents the loss of HRQoL due to illness.

To assess the impact of measles infection on HRQoL, patients must complete the EQ-5D-3L when healthy (at recovery) and for the worst day of infection. We assumed that the QALY loss associated with measles for each individual can be represented by a triangular shape, as shown in Figure 1. A more precise picture would be possible if patients completed the EQ-5D-3L more frequently during their infection. In absence of these data we assume that we can represent the QALY loss as a linear deterioration in HRQoL from a recovery reading to its level on the worst day of infection. As a comparison, we also estimated HRQoL directly using the VAS, with HRQoL given by VAS score divided by 100.

Notification of potential study participants was received by the specialist epidemiologist for measles at Public Health England in Colindale who excluded ineligible patients. Letters and questionnaires were sent using a database updated daily with new notifications of suspected measles cases. In the analysis that follows, we consider only those individuals with laboratory-confirmed measles.

### Anonymised data

All questionnaires sent to confirmed or suspected measles cases were anonymised. A questionnaire was linked to the appropriate follow-up questionnaire using the HP Zone ID, an anonymised ID data field used on Public Health England databases. Sensitive patient identifiers such as the distribution address were handled by Public Health England, whereas the returned and anonymous questionnaires were processed by researchers at the London School of Hygiene and Tropical Medicine, with no links or access to the original sensitive information. All medical records used in the analysis were also anonymised by Public Health England using the HP Zone ID.

### Ethics approval

In accordance with The Health Service (Control of Patient Information) Regulations 2002 No. 1438 Section 251 Regulation 3 [21], Public Health England may process confidential patient information with a view to monitoring and managing

- outbreaks of communicable disease;
- incidents of exposure to communicable disease;
- the delivery, efficacy and safety of immunisation programmes.

### Data analysis

Data were analysed using Microsoft Excel 2007 and R (version 3.0.2) [22]. Public Health England obtained hospitalisation records for individuals who received the questionnaire, so hospitalisation rates were compared between responders and non-responders to test for severity bias. HRQoL data were analysed only for those patients who completed all five dimensions of health on the EQ-5D-3L in addition to reporting the duration of their illness. We calculated the QALY-loss due to measles using the EQ-5D-3L and the VAS and compared the two systems. We examined the three age-specific EQ-5D-3L questionnaires and looked for differences in the QALYs lost due to measles infection. Reported 95% confidence intervals of the means are based on 1,000 bootstrap replications.

The EQ-5D-3L requires the respondent to complete all five dimensions of the classification system in order to calculate a health-state utility. Omitting the response to any of the dimensions means the remaining responses cannot be used for this purpose, therefore a missing-value regression analysis was conducted using the VAS score to estimate the EQ-5D-3L utility where patients had completed the VAS but not all five dimensions of health. When assessing the HRQoL in individuals with haemophilia Miners et al. [23] showed a correlation between EQ-5D-3L utility and the VAS scores (R = 0.67, p<0.0001).

## Results

683 questionnaires were sent; 507 to individuals with confirmed measles and 176 to individuals with unconfirmed/suspected measles. 203 questionnaires were returned from those with confirmed measles (40.0%). 45 questionnaires from individuals with unconfirmed/suspected measles were returned (25.6%). From the 203 individuals with confirmed measles who returned their first questionnaires we received 63 follow-up questionnaires (31.0%). 103 of the returned first questionnaires had been completed after recovery from measles so the HRQoL measurement on the day of completion could be used as the recovery HRQoL measurement.

### Demographic and vaccination data

**Figure 2:**
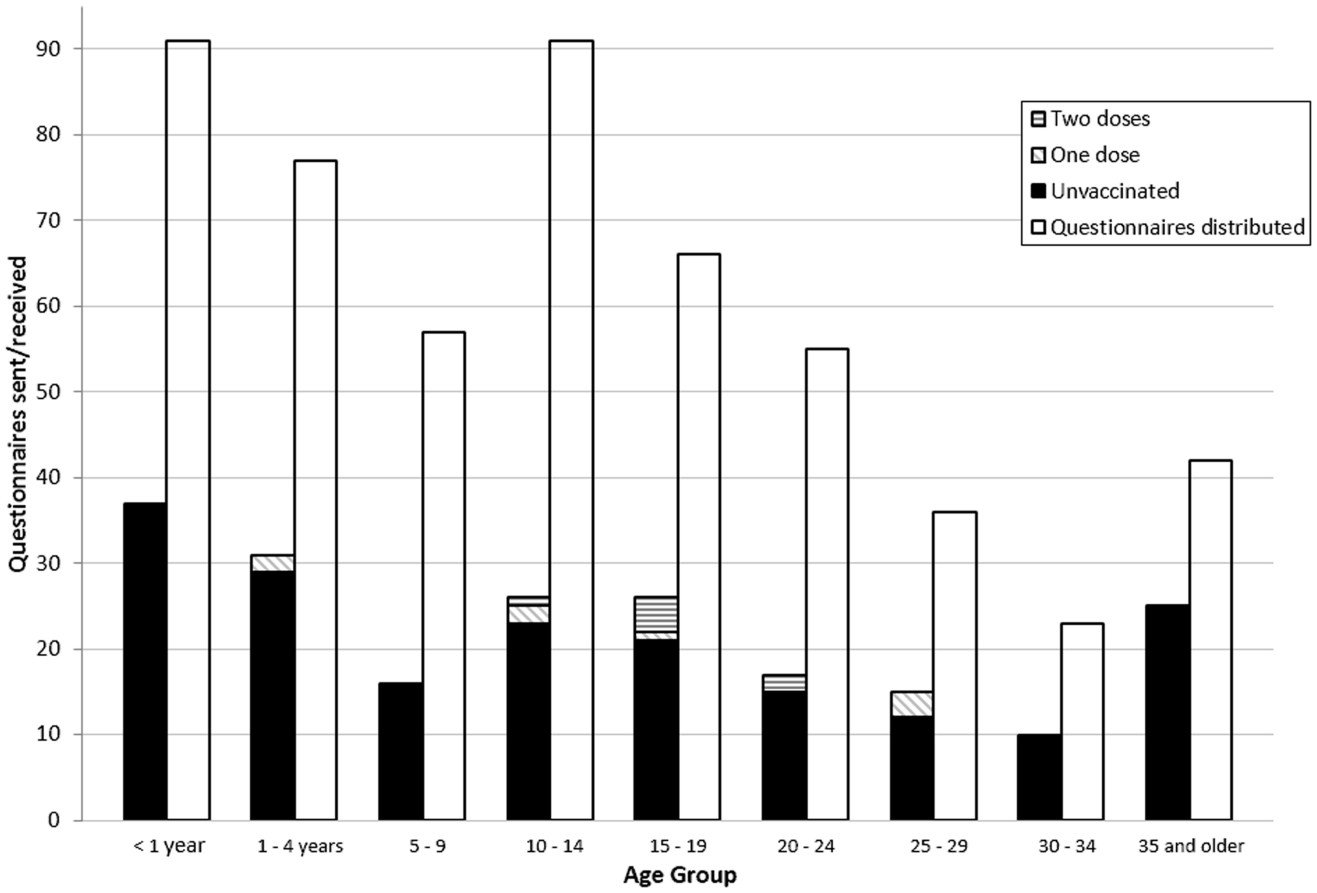
Age-specific distribution of questionnaires sent, questionnaire response, and vaccination status for confirmed cases of measles. The right columns of each age group show the age-distribution of questionnaires sent to confirmed measles cases (left axis); the left columns show the age-distribution of responses (left axis), split into those who are have received no MMR vaccinations (black), one MMR vaccination (diagonal stripe) and two MMR vaccinations (horizontal stripe).

101 (49.8%) of the 203 responses were from female patients. 68 (33.5%) of the respondents were under five years old (Figure 2). 188 (92.6%) had not yet received their first dose of the MMR vaccine. The age distribution of those individuals who returned the questionnaire was similar to the age distribution of confirmed measles cases invited to participate (Figure 2).

### Severity bias

Among the 507 individuals with confirmed measles to whom questionnaires were sent, Public Health England could not obtain hospitalisation records for 20 individuals (3.9%) as their GP's database had not been updated with any details of potential hospitalisations post-notification. Of the remaining individuals, 75 of the 199 individuals who were hospitalised returned their questionnaire and 120 of the 288 individuals not hospitalised returned theirs. We found that there was no evidence that hospitalised individuals were more likely to return the questionnaires (χ^2^ = 0.78 and p = 0.38).

**Figure 3:**
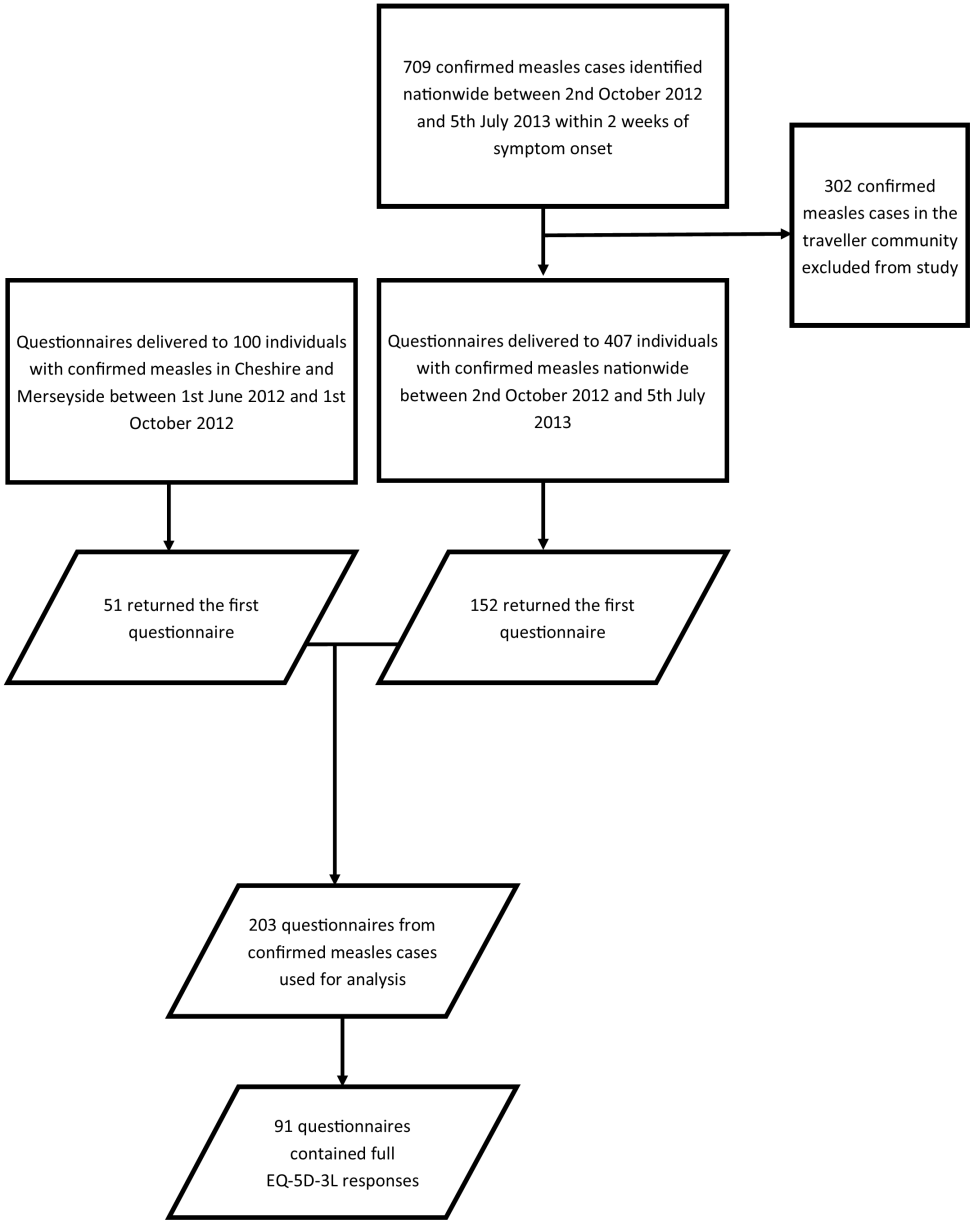
Flow chart for the study showing the number of questionnaires that were distributed to confirmed measles cases; the number of questionnaires returned for analysis; and the number of questionnaires returned for analysis that included the necessary information for EQ-5D-3L health utilities to be calculated.

The remaining results refer only to the 203 questionnaires returned by individuals with confirmed measles. Figure 3 shows the number of questionnaires sent and eventually used in the analysis.

### Impact at home

128 (63.1%) individuals with confirmed measles reported spending time off work or school due to measles infection (Table 1), of whom those who had fully recovered reported a mean time spent at home of 9.6 days (95% CI: 9.3 – 11.7). 75 (36.9%) individuals with confirmed measles reported that a caregiver spent time away from work during their infection, of whom those who had fully recovered reported a mean time spent away from work by carers of 7.3 days (95% CI: 5.7 – 7.9). 74 (36.5%) individuals reported spending at least one night in hospital, of whom those who had fully recovered reported a mean length of stay of 4.2 nights (median 4.0 nights). The median worst day of perceived symptoms was the fifth day and the mean duration of perceived symptoms was 13.8 days (95% CI: 12.6 – 15.1).

**Table 1:** Impact of measles infection.

	Among confirmed measles cases (n = 203)	Age under 7 years (n = 70)	Age 7–12 years (n = 25)	Age 13 years and over (n = 108)
Worst day (median, mean, mode)	5, 5.61, 4	5, 5.58, 5	6, 7.56, 5	5, 5.15, 4
Mean duration of perceived symptoms (95% CI)	13.8 days (12.6 – 15.1)	12.8 days (11.0 – 14.9)	13.5 (10.4 – 17.1)	14.4 (12.7 – 16.2)
Individuals reporting time off work or school	128 (63.1%)	26 (37.1%)	22 (88.0%)	80 (74.1%)
Mean time off work or school for patients (95% CI)	9.6 days (9.3 – 11.7)	8.6 days (6.8 – 10.5)	9.1 days (7.4 – 10.8)	10.1 days (8.8 – 11.5)
Individuals reporting time off work for primary caregivers	75 (39.6%)	31 (44.3%)	10 (40.0%)	34 (31.5%)
Mean time off work for primary caregivers (95% CI)	7.3 days (5.7 – 7.9)	7.0 days (4.9 – 9.2)	7.7 days (4.3 – 11.3)	7.2 days (5.0 – 9.5)
Individuals reporting at least one night in hospital	74 (36.5%)	23 (32.9%)	2 (8.0%)	49 (45.4%)
Number of nights spent in hospital (median, mean, mode)	4.0, 4.2, 1.0	3.0, 4.0, 1.0	4.0, 4.0, 4.0	4.0, 4.4, 1.0

The mean time off work or school for patients and for primary caregivers is the mean time for those who reported at least one day of absence. Likewise, the number of nights in hospital applies only to those individuals who reported at least one night in hospital. 95% confidence intervals of the mean are based on 1,000 bootstrap replications. The first column shows results for the whole sample; the subsequent 3 columns split the sample into the three age groups considered.

### Contact with the health services

193 (95.1%) individuals with confirmed measles reported at least one contact with the health services. The remaining 10 individuals may have come to the attention of the local Health Protection Unit (HPU) through contact tracing of another confirmed measles case or may have been reported directly to the HPU by a teacher, parent or guardian, thereby not having any contact with the health services before their case status was confirmed. The median number of contacts with the health services was 3.0 during the period of infection but this was highly skewed with a mean of 4.0 and 5 people having more than 10 contacts. The mean time between perceived symptom onset and first contacting the different local health services was about 3.6 days irrespective of which service (NHS Direct, GP, A&E, etc.) was first contacted.

### EQ-5D-3L dimensions results

91 of the 203 confirmed measles cases completed all five dimensions of health for the EQ-5D-3L on the worst day of infection and after a full recovery from measles infection and reported the duration of perceived symptoms, thus enabling the calculation of QALYs lost. On the worst day of infection, these individuals reported their health according to each of the EQ-5D-3L dimensions of health as shown in Table 2.

**Table 2:** Responses to each dimension of health for the worst day of infection for individuals with confirmed measles who provided the full data set to facilitate the calculation of QALY loss associated with measles.

	Evaluating HRQoL on the worst day of infection (n = 91)		
EQ-5D dimensions of health	No problems	Some problems	Severe problems
Mobility	10 (11.0%)	35 (38.5%)	46 (50.5%)
Self-care	20 (22.0%)	35 (38.5%)	36 (39.6%)
Usual activities	3 (3.3%)	17 (18.7%)	71 (78.0%)
Pain or discomfort	9 (9.9%)	45 (49.5%)	37 (40.7%)
Anxiety or depression	33 (36.3%)	34 (37.4%)	24 (26.4%)

194 of the 203 individuals with confirmed measles (95.6%) who returned a questionnaire also returned a completed VAS.

### HRQoL results

The overall QALY loss, calculated using the EuroQol EQ-5D-3L, associated with measles was 0.019 QALYs per patient (95% CI: 0.016 – 0.022), the equivalent of 6.9 QALDs per patient (95% CI: 5.84 – 8.02) (Table 3).

**Table 3:** Impact on HRQoL of measles for the 91 individuals with confirmed measles for whom QALY loss could be calculated using the EQ-5D-3L.

	Health-related quality of life results (n = 91)	Age under 7 years (n = 15)	Age 7–12 years (n = 18)	Age 13 years and over (n = 58)
EQ-5D Baseline HRQoL (95% CI)	0.96 (0.93 – 0.98)	0.89 (0.78 – 0.98)	0.98 (0.96 – 1.00)	0.94 (0.91 – 0.97)
EQ-5D Worst day HRQoL (95% CI)	0.00 (−0.09 – 0.09)	−0.26 (−0.43 – −0.08)	−0.07 (−0.22 – 0.10)	−0.05 (−0.13 – 0.04)
VAS Background (95% CI)	92 (90 – 94)	93 (91 – 95)	91 (87 – 95)	89 (86 – 91)
VAS Worst day (95% CI)	21 (17 – 24)	18 (14 – 22)	19 (13 – 25)	19 (15 – 23)
Overall QALY loss (95% CI)	0.019 (0.016 – 0.022)	0.017 (0.012 – 0.022)	0.020 (0.014 – 0.028)	0.019 (0.016 – 0.023)
Overall QALD loss (95% CI)	6.90 (5.84 – 8.02)	6.29 (4.51 – 8.11)	7.28 (5.07 – 10.10)	6.94 (5.73 – 8.33)

95% confidence intervals of the mean are based on 1,000 bootstrap replications. The first column shows results for the whole sample; the subsequent 3 columns split the sample into the three age groups considered.

### HRQoL through the VAS

The overall QALY loss associated with measles using the VAS score was the equivalent of 4.92 QALDs (95% CI: 4.15 – 5.86) or 0.013 QALYs (95% CI: 0.011 – 0.016). There is very strong evidence that the VAS gives different results when compared to the EQ-5D-3L algorithm using the paired Wilcoxon test (V = 649, p<0.0001) using the HRQoL results from the 91 individuals with confirmed measles who completed all aspects of the EuroQol EQ-5D questionnaire.

### Overall burden of regional epidemics

Public Health England reported that there had been 2,366 laboratory-confirmed cases of measles in England for twelve months from 1^st^ June 2012, the beginning of the study period [24], [25]. Using our estimates above for the burden of measles infection, the age-adjusted overall burden of disease in this period was approximately 16,164 QALDs (95% CI: 15,740 – 16,645), or 44.2 QALYs (95% CI: 43.2 – 45.6). 1,534 of these confirmed cases would have taken time off work or school, resulting in 14,527 age-adjusted days of lost productivity (95%CI: 14,215 – 14,848). When including primary caregivers taking time off work, a further 904 people would have taken time off work resulting in an age-adjusted total number of 23,110 days of lost productivity (95%CI: 22,661 – 23,522). 95% confidence intervals of the mean are based on 1,000 bootstrap replications.

### Missing data analysis

Each patient was sent a maximum of three EQ-5D-3L questionnaires: for the worst day of infection, for the date that the first questionnaire was received and the recovery HRQoL reading. From a maximum of 744 eligible questionnaires from both individuals with confirmed measles and individuals with unconfirmed/suspected measles, 397 contained both a EQ-5D-3L questionnaire with responses to all dimension of HRQoL and a completed VAS score.

Assuming that EQ-5D responses were missing at random and that the VAS score can be used to predict missing EQ-5D utilities, we used a multiple imputation method through the Amelia II statistical package in R [26] to impute EQ-5D utilities where the individual had completed the VAS. This added 26 more observations and the overall QALY loss from the interpolated data was equivalent to 6.81 days (95% CI 5.68 – 8.04), very similar to the QALY and equivalent QALD loss from non-interpolated data reported in Table 3.

### Implications for missing HRQoL data

49 (70%) of the 70 EQ-5D proxy questionnaires for children aged under 7 years for the worst day of infection returned were missing in the self-care dimension (Table 4). This suggests that parents or guardians have difficultly completing this dimension of the EQ-5D-3L as a proxy for their young children. 28 of the returned EQ-5D proxy questionnaires (40%) did not have a response recorded in the mobility dimension. Fewer missing responses were returned for the remaining three dimensions.

**Table 4:** Number of missing responses to each EQ-5D dimension of health on the worst day of infection.

	Responses	Complete responses	Missing: Mobility	Missing: Self-care	Missing: Usual activities	Missing: Pain	Missing: Depression
**EQ-5D proxy**	70	20 (28.6%)	28 (40.0%)	49 (70.0%)	16 (22.9%)	8 (11.4%)	12 (17.1%)
**EQ-5D-Y**	25	25 (100.0%)	0 (0.0%)	0 (0.0%)	0 (0.0%)	0 (0.0%)	0 (0.0%)
**EQ-5D**	108	102 (94.4%)	5 (4.6%)	3 (2.8%)	3 (2.8%)	2 (1.9%)	2 (1.9%)
	**203 (100%)**	**147 (72.4%)**					

None of the 25 EQ-5D-Y questionnaires for children aged 7 – 12 years had a missing response for any of the five dimensions on the worst day of infection. Few EQ-5D-3L questionnaires for individuals aged 13 years and older had missing responses for the dimensions of health: 5 questionnaires (4.6%) were missing a response in the mobility dimension, with fewer missing responses in the remaining dimensions.

### Measuring HRQoL using age-specific EQ-5D-3L

The standard EQ-5D-3L was used by individuals aged 13 years and older. The mean QALD-loss attributable to measles for this group was 6.9 days (95% CI: 4.9 – 9.1). For individuals aged between 7 – 12 years the EQ-5D-Y was used to report a mean QALD-loss of 7.3 days (95% CI: 3.7 – 13.0). For infants aged under 7 years the EQ-5D (proxy) was used to report a mean QALD-loss of 6.2 days (95% CI: 3.6 – 9.0). Using the independent Mann-Whitney test there was no evidence that the measured HRQoL loss is dependent on the EQ-5D-3L questionnaire used (W = 483.5 and p = 0.64 when compared to EQ-5D-Y; W = 433.5 and p = 0.99 when compared to EQ-5D proxy).

## Discussion

We have used the confirmed measles cases reported since June 2012 to calculate the short-term impact on HRQoL of measles, with measurements taken during the 2012-13 regional measles epidemics in England. We found that measles infection causes a short-term QALY-loss of 0.019 QALYs, or 6.9 QALDs, per patient, with perceived symptoms lasting 13.8 days. For context, the short-term impact on HRQoL of H1N1v influenza was 0.008 QALYs, or 2.92 QALDs, per patient [19]. The impact on HRQoL of natural varicella was 0.0027 QALYs, or 0.99 QALDs (<15 years old) [27] and 0.0038 QALYs, or 1.39 QALDs (≥15 years old) per patient [28].

To our knowledge this is the first attempt to calculate the impact on HRQoL of measles infection. This study was a patient-based retrospective study that invited all eligible confirmed cases of measles since mid-2012 in the general population to participate. The response rate was reasonable for a postal survey with a return rate of 40%. In addition to quantifying the short-term impact of measles on HRQoL we have also described the wider impact in terms of time off work or school for individuals with measles and their primary caregivers.

With MMR coverage still below the herd immunity threshold, the potential for further measles outbreaks still exists within England. Following this study, cost-effectiveness analyses for possible interventions for such outbreaks may now be performed using QALYs, so that a single generic metric is compared across all analyses.

Using the VAS to derive health QALDs underestimates the impact of measles infection on HRQoL in comparison to the EQ-5D-3L, according to our sample of individuals with confirmed measles. Indeed, the VAS is not a preference-based system so it should not be used alone to calculate QALYs [29].

55.2% of individuals with confirmed measles who returned their questionnaires did not provide all of the data necessary to calculate QALY loss associated with measles infection. Completion was poor for the EQ-5D proxy version administered to parents or guardians to complete on behalf of a child aged less than 7 years; 70% of returned EQ-5D proxy questionnaires had a missing response to the self-care dimension (Table 4). This is hardly surprising, since it is unclear how one ought to answer such a question, but it means that the proxy form of the EQ-5D-3L may not be appropriate for evaluating a young child's HRQoL. Fewer completion issues were evident with the EQ-5D-3L for individuals with confirmed measles aged 13 and older, and no completed issues were evident for the EQ-5D-Y administered to children aged 7 – 12 years. In contrast to the missing responses to the questions about health dimensions, 95.6% of individuals with confirmed measles who returned a questionnaire also returned a completed VAS; this suggests that individuals with confirmed measles found it easier to complete the VAS than the EQ-5D-3L dimensions.

We found that estimated HRQoL loss is not dependent on the EQ-5D-3L questionnaire used, i.e. the EQ-5D proxy and EQ-5D-Y give similar values of HRQoL when compared to the standard EQ-5D-3L. However, we note that both the EQ-5D-Y and EQ-5D proxy questionnaires currently use the same scoring tariff as the EQ-5D-3L. That is, the value of different health states measured by the EQ-5D-3L is assumed to be identical for all respondents in our study. This assumption has been challenged in the past [30]-[32] and EuroQol are currently developing a child-specific tariff for the EQ-5D-Y.

We note that in the regional epidemics in Cheshire and Merseyside only 18% of confirmed cases were hospitalised [11], in comparison to 36.5% of our sample reporting spending at least one night in hospital, though the authors of that study suggested that the hospitalisation data from that study may underestimate the true rate. From our sample of confirmed cases we did not find evidence that the more severe cases were more likely to respond to our questionnaire.

### Limitations

This study was a retrospective evaluation of the impact of measles infection on short-term HRQoL, using self-reported metrics. It would be preferable to evaluate short-term HRQoL loss in a controlled environment with daily EQ-5D-3L questionnaire completion and additional laboratory confirmation of items such as onset date and duration of infection. However, our study protocol followed similar evaluations of HRQoL loss for other infectious disease and was successfully designed and executed during a nationwide measles outbreak.

We assumed that the deterioration in HRQoL is linearly related to the duration of infection (Figure 1) and used a triangular shape to describe the QALY loss. This assumption could be tested if HRQoL were measured more often over the course of measles infection, providing sufficient information to gauge how HRQoL varies during infection. However, this proposal may be infeasible as it places a larger burden on the individuals with measles during their period of infection. When Hollmann et al. (2013) [33] calculated the impact of H1N1 influenza on patients in Spain they assumed that the health utility corresponding to the worst day of infection is experienced constantly throughout infection. This assumption means that HRQoL drops to its lowest possible level from day one of infection and returns to its highest level upon recovery. In comparison to our method, this doubles the impact on HRQoL.

Individuals were unlikely to complete the EQ-5D-3L for the worst day of their illness on that day, as we were unable to send questionnaires to individuals until after confirmation of measles was received, which was likely to be after the worst day of illness. This may be a source of recall bias but we attempted to minimise this by sending questionnaires to confirmed cases as quickly as possible. The median time between the perceived symptoms onset and the date of completing the questionnaire was 12.0 days (mean 16.8 days, mode 5.0 days). Using the independent Wilcoxon test we found no evidence that the short-term impact on HRQoL was associated with the length of time between perceived symptoms onset and the date of completion of the questionnaire. Those individuals completing the questionnaire within one week of perceived symptoms onset reported a mean QALD-loss of 7.88 days (95% CI: 5.1 – 11.92), as compared to those completing the questionnaire between 8 – 14 days (5.64 QALDs, 95% CI: 3.03 – 8.16, W = 200 and p = 0.29) and to those completing the questionnaire more than 14 days after symptom onset (6.03 QALDs, 95% CI: 4.30 – 8.12, W = 543 and p = 0.19).

10 patients reported that they did not have any contact with the health services before their case status was confirmed. This may be because they were already known to the local HPU through contact tracing of another confirmed case or were separately reported to the HPU without contacting the health services. However, we recognise that they may have failed to report a contact with the health services before notification to the HPU and therefore could be a source of misclassification bias in our study.

In our calculation of QALY loss due to measles we used the reported perceived length of symptoms rather than duration of illness as obtained through serology. However, we feel that this assumption and use of a proxy is justified as an individual will only report a lower health state to their preferred health state when their symptoms affect their wellbeing; thus perceived symptoms are the relevant factor.
